# Exploration of Alkaliphilic Bacteria *Alkalitalea saponilacus* Identifies Glycoside Hydrolases With Biotechnological Potential

**DOI:** 10.1111/1751-7915.70448

**Published:** 2026-09-21

**Authors:** A. Stephen, N. L. Brown, R. Rostron, R. J. Wigham, A. K. Malekpour, G. W. Black, A. L. Morales Garcia, H. C. L. Yau, N. J. Lant, J. Munoz‐Munoz

**Affiliations:** ^1^ School of Geography and Natural Sciences Northumbria University Newcastle upon Tyne UK; ^2^ Department of Biosciences Durham University Durham UK; ^3^ Procter and Gamble Newcastle Innovation Centre Newcastle upon Tyne UK

**Keywords:** alkaline detergent enzymes, extremophiles, glycoside hydrolase, soda lakes, stain removal, whiteness maintenance

## Abstract

Soap Lake, a small meromictic lake in Washington State, USA, harbours a diverse microbial community adapted to high carbonate, sulfate and sulfide concentrations and a pH of approximately 9.8. *Alkalitalea saponilacus*, an anaerobic obligate bacterium from the lowest layer of Soap Lake, encodes multiple secreted enzymes, bioinformatically annotated as active on a range of carbohydrates. The ability of these enzymes to function under their extreme native conditions offers considerable industrial potential, most notably in the production of granular laundry and automatic dishwashing detergents that require robust performance under highly ionic conditions. Whilst some alkaline‐stable enzyme classes exist within current detergent formulations, there is a significant underrepresentation of non‐starch polysaccharide‐targeting candidates, positioning *A. saponilacus* as an untapped reservoir for this application. This study investigates *A. saponilacus* beta‐glucan degradation and confirms, using RT‐qPCR, the involvement of polysaccharide utilisation loci (PUL) 10 in this process. A representative glycoside hydrolase 16 from this PUL has been heterologously expressed and evaluated as a beta‐glucan‐targeting candidate, stable under harsh alkaline conditions. To demonstrate the industrial potential of *A. saponilacus* across both genomic location and functional enzyme families, a mannan‐targeting glycoside hydrolase 26 and a cellulose‐targeting glycoside hydrolase 9 were also evaluated for substrate deconstruction under high pH conditions. All three model GHs were tested for complex stain removal within a commercial detergent formula, where they exhibited considerable potential as additives. These results reinforce the prospects of the respective molecules, and ultimately the *A. saponilacus* proteome, for biotechnological applications where performance under alkaline or strongly ionic conditions is required.

## Graphical abstract

Soda lakes represent an excellent resource in the search for microbes adapted to alkaline and hypersaline conditions. In this study we demonstrate the ability of *Alkalitalea saponilacus* to degrade multiple carbon sources representing a promising bacterium for biotechnological applications where performance under alkaline or strongly ionic conditions is required.

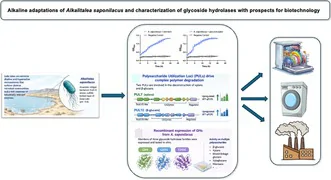

## 1 Introduction

Soda lakes represent some of the most unique environments on the planet, characterised by extreme alkalinity, salinity and ionic concentrations, with pH generally ranging from 9 to 12. These geographical features form in arid regions when evaporation exceeds precipitation, causing a build‐up in sodium carbonate and bicarbonate salts from dissolved volcanic rock (Ren and Wang 2025). The strongly ionic environment created by this is widely inhospitable to life; however, specialised alkaliphilic organisms thrive under such conditions. As a result of various geological factors including volcanic activity, rock weathering and restricted drainage basins, each soda lake is distinct in its chemical and biological characteristics (Boros and Kolpakova 2018). This makes every soda lake a valuable, individual resource in the identification and biochemical understanding of bacteria adapted to extreme environments. Because their physiochemical properties resemble those hypothesised for certain extraterrestrial settings, such as ancient Martian basins or subsurface oceans on icy moons, soda lakes are also considered valuable analogues for astrobiological studies (Jangir and Dutta 2026). This combination of geochemical and ecological uniqueness is exemplified by Soap Lake in Washington State (Vanderlaan et al. 2025).

Soap Lake, located in central Washington State (USA), is a highly alkaline, meromictic soda lake with complex physicochemical stratification and specialised microbial communities. This relatively small lake is permanently divided into two main layers: a mixolimnion (surface waters down to about 19 m) that is relatively warm (11°C–21°C), brackish, and well oxygenated, and a monimolimnion (21–27 m) that is colder (6°C–10°C), anoxic, hypersaline, and contains unprecedentedly high concentrations of dissolved sulfide that have been reported to exceed 150 mM (Vanderlaan et al. 2025). Superimposed on this vertical structure, Soap Lake's waters are enriched in sodium carbonate, bicarbonate and sulfate, generating steep gradients in salinity, redox potential and sulfur chemistry that strongly influence habitat suitability. Consequently, the microbial communities inhabiting the two layers differ markedly, with the upper mixolimnion dominated by Pseudomonadota (especially Alphaproteobacteria), Actinomycetota and Bacteroidota, while the lower anoxygenic monimolimnion is dominated by Bacillota adapted to hypersaline, sulfidic conditions. Over time, this unusual combination of geochemistry and stable stratification has fostered diverse microbial assemblages that are now used as model systems for studying life at the limits of alkalinity, salinity and sulfide. Today, Soap Lake serves as an important interdisciplinary research site for probing microbial resilience, geochemical processes and the biotechnological potential of extremophiles: a key example of which is alkaliphilic bacterium *Alkalitalea saponilacus*, isolated from the monimolimnion (Zhao and Chen 2012; Vanderlaan et al. 2025).

*Alkalitalea saponilacus* is an alkaliphilic and moderately halophilic bacterium that represents a key member of the microbial community inhabiting the extreme environment of Soap Lake (Zhao and Chen 2012). First isolated from the alkaline waters and sediments of this lake, *A. saponilacus* belongs to the family Marinilabiliaceae within the phylum Bacteroidota, a lineage that includes many organisms adapted to specialised ecological niches (Zhang et al. 2025). This bacterium thrives under high‐pH and saline conditions, reflecting physiological and metabolic adaptations that enable survival where few other microorganisms can persist. Morphologically, *A. saponilacus* is rod‐shaped and exhibits obligate anaerobic metabolism, utilising a broad range of carbohydrates, indicating its importance for the degradation of organic matter and nutrient cycling in soda lake ecosystems (Liao et al. 2018). By contributing to the breakdown of complex organic substrates in such extreme habitats, *A. saponilacus* exemplifies how microbial specialisation underpins ecosystem functioning and sets the stage for the deployment of diverse carbohydrate‐active enzymes that mediate these processes.

Carbohydrate‐active enzymes (CAZymes) produced by *A. saponilacus* are adapted to retain catalytic activity under the extreme alkaline and saline conditions characteristic of soda lakes such as Soap Lake (Jeilu et al. 2024). Alkaline‐adapted enzymes often display structural modifications, including altered surface charge distributions and enhanced stabilisation of their active sites, which promote proper folding and function in environments that would denature most conventional proteins (Jeilu et al. 2026). Such adaptations are particularly important for secreted CAZymes, which must remain active in the extracellular milieu where pH and ionic strength are especially extreme. At the genomic level, the organisation and regulation of CAZyme‐encoding genes in *A. saponilacus* reflect selective pressures to efficiently sense, access and degrade complex polysaccharides under these harsh conditions. Together, these molecular and genomic features underpin the organism's specialisation for carbon turnover in soda lakes and provide the biochemical foundation for the potential technological exploitation of its CAZymes in applied settings (Jeilu et al. 2022).

Enzymes from *A. saponilacus* therefore hold considerable promise for diverse biotechnological applications that require biocatalysts functioning under highly alkaline and saline conditions. In detergent formulations, CAZymes that maintain activity in carbonate‐rich, high‐pH solutions could enhance the removal of polysaccharide‐containing soils under harsh washing regimes, reducing energy‐intensive wash requirements (Mesbah 2022). This is complemented by current efforts in cold‐adapted enzyme development, which also enable effective low‐energy washing (Oliva et al. 2024). In lignocellulosic biomass deconstruction and biofuel production, the robustness of alkaline enzymes at elevated pH levels and ionic strength could improve process efficiency by enabling more aggressive pretreatment conditions without loss of enzymatic activity (Kim et al. 2016). In waste management and environmental remediation, such enzymes could facilitate the breakdown of complex organic matter in alkaline industrial effluents or engineered treatment systems, contributing to more sustainable and energy‐efficient processing (Feng et al. 2025). However, despite this apparent potential, the CAZyme repertoire of *A. saponilacus* and the biochemical properties of representative enzymes under extreme alkaline conditions remain poorly characterised, limiting their rational exploitation in industrial and environmental applications and therefore motivating the investigations carried out in this study.

This study aims to further investigate alkaline‐tolerant bacteria *Alkalitalea saponilacus* and its encoded CAZymes. The nature of carbohydrate metabolism by this bacterium has been studied and the predicted function of a beta‐glucan‐targeting polysaccharide utilisation loci 10 has been confirmed. The industrial application of the *A. saponilacus* proteome has been validated via the recombinant expression of a glycoside hydrolase family 16 (GH16), 26 and 9. Modern granular laundry and automatic dishwashing detergents operate at alkaline pH (typically pH 9.5–11.0) and contain high carbonate and surfactant concentrations, inactivating most mesophilic enzymes. Therefore, enzymes which retain catalytic activity, structural stability and substrate accessibility under these conditions are significantly commercially relevant. This study reports respective enzymes GH16, GH26 and GH9 exhibit strong through‐the‐wash stain removal and whiteness performance under harsh detergent conditions. This highlights the significant potential of haloalkaline bacterium *A. saponilacus* CAZymes as a promising source for detergent compatible enzymes.

## 2 Materials and Methods

### 2.1 Growth Media and Reagents

Bacterial strains and plasmids utilised in this study are listed in Table S1. *Alkalitalea saponilacus* DSMZ 24412 (*A. saponilacus*) was cultivated in MAB medium (DSMZ:1357) supplemented with 0.5% glucose (w/v) (Sigma‐Aldrich, UK), and incubated anaerobically in a modular atmosphere‐controlled system (Davidson and Hardy, UK) at 37°C. *Escherichia coli* strains were routinely cultivated in BD Difco lysogeny broth (LB; BD, UK) at 37°C with agitation at 200 rpm. Where appropriate, the growth medium was supplemented with kanamycin to a final concentration of 50 μg/mL.

Carbohydrate utilisation by *A. saponilacus* DSMZ 24412 was examined in MAB medium supplemented with the specified carbohydrate (0.5%, w/v). Assessed carbohydrates are described in Table S2 and were obtained from Sigma Aldrich (laminarin, barley β‐glucan, pectin, carboxymethyl cellulose (CMC), gum arabic and alginate) or Megazyme (birchwood xylan, wheat arabinoxylan, tamarind xyloglucan). Monosaccharides were all obtained from Sigma‐Aldrich (UK).

Cell turbidity was monitored via the measurement of optical density at 600 nm (OD_600_). To quantify bacterial growth dynamics, 5 mL of freshly prepared MAB medium supplemented with the designated carbohydrate (0.5%, w/v) was inoculated with 50 μL of an overnight culture grown in MAB + 0.5% (w/v) glucose to an OD_600_ of approximately 1. Uninoculated MAB medium was used as a negative control. Cultures were incubated anaerobically at 37°C and turbidity, measured via OD_600nm_, was continuously measured for 24 h using a spectrophotometer inside the anaerobic cabinet. All assays were conducted in triplicate.

### 2.2 Transcriptome Analysis

*A. saponilacus* was grown overnight in MAB + 0.5% (w/v) glucose and then subcultivated in MAB + 0.5% (w/v) of either glucose, xylan, or laminarin to an OD_600nm_ of 0.5–0.8. Four millilitres of each culture were centrifuged (Dlab D3024R) at 5500 *g* for 4 min. Bacterial cultures were harvested in triplicate at mid‐log phase [OD_600_, ~0.6] after 5 h of incubation, placed in RNA protect (Qiagen) for immediate stabilisation of RNA, and then stored at −20°C. RNA was extracted and purified with the RNeasy minikit (Qiagen), and RNA purity was assessed via spectrophotometry. 1 μg of RNA was used for reverse transcription and synthesis of cDNA (SuperScript VILO master mix; Invitrogen). Quantitative PCRs (20 μL final volume) using specific primers were performed with a SensiFast SYBR Lo‐ROX kit (Bioline) on a 7500 Fast real‐time PCR system (Applied Biosystems) (Table S1). Data were normalised to 16S rRNA transcript levels and changes in expression were calculated as fold change compared to cultures grown in MAB supplemented with glucose or xylose as specified.

### 2.3 Cloning, Expression and Purification of Recombinant Proteins

The genes associated with *As*GH9 (CDL62_13645), *As*GH16 (CDL62_12355) and *As*GH26 (CDL62_12545) were amplified from *A. saponilacus* using its genomic DNA as a template. Amplified products were digested at NdeI and XhoI restriction sites and ligated into a pET28a (Novagen) vector to encode the enzyme of interest with an N‐terminal His_6_ tag (Table S1). For this, *E. coli* TOP10 cells (ThermoFisher Scientific) were used. The recombinant construct was sequenced (Eurofins Genomics) to verify its genetic integrity and then used to transform *E. coli* BL21 (DE3) expression cells (Thermo‐Fisher Scientific). Cells were cultured in Luria‐Bertani (LB) medium containing a final concentration of 50 μg/mL kanamycin to mid‐log phase (OD_600nm_ of ~0.6). Protein expression was induced by adding 0.1 mM isopropyl β‐D‐1‐thiogalactopyranoside (IPTG) to cells which were subsequently incubated at 16°C and 100 rpm overnight. Cells were harvested by centrifugation (4000 *g*) and re‐suspended in TALON buffer (100 mM NaCl, 20 mM Tris/HCl pH 8.0). Resuspended cells were disrupted via sonication and centrifuged (16,000 *g*) for 30 min at 4°C to obtain the cell free extract. Recombinant proteins were then purified using immobilised metal affinity chromatography (IMAC) with Talon, a cobalt‐based matrix. Cell Free Extract (CFE) was loaded onto a column containing Talon resin and washed with Talon buffer. Another wash was performed with Talon buffer containing 10 mM imidazole followed by recombinant protein elution with 100 mM imidazole. Purified proteins were exchanged into the desired buffer by standard dialysis.

### 2.4 Assessment of Enzymatic Activity

All enzyme assays were carried out in 100 mM Sodium Carbonate buffer (Thermofisher, UK), pH 10.0 and performed in triplicate. Assays were carried out at 37°C employing 1 μM of each GH in the presence of 1 mg/mL of each polysaccharide. Aliquots were incubated for 24 h, and product release was assessed using thin‐layer chromatography (TLC) and high‐pressure anion exchange chromatography (HPAEC) with pulsed amperometric detection (PAD). Sugars (mono and short oligosaccharides) were separated on a Carbopac PA1 guard and analytical column in an isocratic programme of 100 mM sodium hydroxide for 40 min and then with a 100% linear gradient of 500 mM sodium acetate over 60 min. Sugars were detected using the carbohydrate standard quad waveform for electrochemical detection at a gold working electrode with an Ag/AgCl pH reference. In addition to HPAEC, TLC was used to visualise the product profile of each enzyme. Aluminium foil TLC plates (Silicagel 60, 20 × 20, Merck) were cut to a minimum height of 10 cm, spotted with 2 μL of end‐point reaction supernatant and allowed to completely dry. TLC plates were placed in running buffer (1‐butanol/acetic acid/water 2:1:1 (v/v)) until the solvent reached 2 cm from the top of the plate. Plates were then removed and dried before visualisation of sugars was obtained via immersion of the TLC plate in Orcinol stain. Plates were dried and developed through heating between 50°C and 80°C.

### 2.5 Assessment of pH‐Profile of Recombinant Enzymes

Enzymatic assays were performed to measure the pH profile of each individual GH family. Polysaccharide hydrolysis was determined using a DNSA (3,5‐dinitrosalicylic acid) reducing‐sugar assay (Miller 1959). Enzyme assays were completed in a final volume of 1 mL in various buffers, (according to the required pH) at 37°C for 2 h. Citrate buffer (0.1 M) was used for pH 3–6, sodium phosphate (0.1 M) for pH 6–8, Tris–HCl (0.1 M) for pH 8–9 and sodium carbonate (0.1 M) for pH 9–12. Substrate and enzyme concentrations were tailored to each individual enzyme in order to best capture initial rate of reaction. Lichenan (1 mg/mL) was used as substrate for AsGH9 (0.5 μM enzyme), curdlan (2 mg/mL) for AsGH16 (0.2 mM enzyme) and glucomannan (2 mg/mL) for AsGH26 (0.2 mM enzyme). Reaction aliquots were taken at set points and added to DNSA reagent in a 1:1 ratio, then heated at 96°C for 10 min. Following heating, and cooling for 10 min, absorbance at 540 nm was recorded using a spectrophotometer and product release was determined using a standard curve of either glucose (*As*GH9, *As*GH16) or mannose (*As*GH26).

### 2.6 Bioinformatics

3‐D structures of *As*GH9, AsGH16 and AsGH26 were modelled using the AlphaFold3 server. Structures were visualised using Pymol (The PyMOL Molecular Graphic system, version 2.0 Schrodinger LLC). Catalytic residues were identified using Expasy ProSite alongside solved structure overlaying (de Castro et al. 2006). Isoelectric point and net charge of proteins across pH range were identified using APBS‐PDB2PQR (Jurrus et al. 2018). The PULDB database was used to predict the PUL composition in *A. saponilacus*.

Lysine content, disulfide bond content, hydrogen bonding network extent, and hydrophobic core packing were assessed for AsGH9, AsGH16 and AsGH26. Signal peptides were manually removed according to SignalP prediction, prior to structural analysis (Teufel et al. 2022). Lysine residues were counted directly from the mature (signal‐peptide‐removed) sequence and expressed as both a raw count and a percentage of total mature sequence length. Cysteine sulfur (SG) atoms were identified in PyMOL, and disulfide bonds were assigned where paired SG atoms fell within 2.5 Å of one another. Backbone hydrogen bonds were identified using DSSP (Hekkelman et al. 2025). Total hydrogen bonds were calculated using the HydrogenBondAnalysis module in MDAnalysis, applying a donor–acceptor distance cutoff of 3.5 Å and a donor–hydrogen–acceptor angle cutoff of 120° (Michaud‐Agrawal et al. 2011; Gowers et al. 2016).

Buried hydrophobic surface area was calculated using FreeSASA applying default reference solvent‐accessible surface area values (Mitternacht 2016). Hydrophobic residues were defined as alanine, valine, leucine, isoleucine, phenylalanine, methionine and tryptophan. The percentage buried was calculated as (1 − mean relative solvent accessibility) × 100, averaged across all hydrophobic residues in each structure. Internal cavity volume was calculated using CASTp (Tian et al. 2018). For each enzyme, pockets containing catalytic residues were excluded from reported cavity volume, as these represent functional substrate‐binding space rather than intradomain packing. Pockets formed due to low‐confidence regions surrounding cleaved signal peptides were also excluded. In the case of *As*GH16, the interdomain cleft characteristic of its multi‐domain architecture was also excluded in cavity volume calculations.

### 2.7 Fabric Washing

For whiteness benefit assessment, whiteness is defined as the restoration or maintenance of the original white appearance of textiles that have acquired a greyish or yellowish hue through repeated use and washing. Knitted cotton fabric (5 cm^2^ swatches) and clean cotton ballast fabrics totalling 30 g were washed in an automated tergotometer (HAHN Automation Group UK, Washington, UK) at 30°C for 30 min, 300 rpm agitation in water measuring 8.7 US grains per gallon hardness, in the presence of 0.04 g/L Carbon black (Alfa Aesar Chemicals, UK, Product 45527.95, 100% compressed) and 2.4 mL/L UK Fairy non bio liquid laundry detergent (pH 8) (batch code: 5098030350 02:50) with or without 2 parts per million (PPM) *As*GH9. Fabrics were then rinsed with water for 5 min at 15°C. This procedure was repeated a further three times and knitted cotton fabric swatches left to dry overnight at ambient room temperature. The colour of fabrics was evaluated before and after washing using the Whiteness index CIE L*a*b* assessment system measured on a Konica Minolta CM‐3610A device and used to calculate the change in Whiteness index between unwashed (pre‐wash) and washed fabrics (Billmeyer 1988).

For make‐up stain removal assessment, 250 μL of Living Nature Foundation Pure Taupe (Living Nature, New Zealand, Batch 220129) was pipetted onto a clean 5 cm^2^ knitted cotton fabric (CW120, 2M Equest UK) using a positive displacement pipette. This stain was then rubbed into the fabric using a silicon applicator until homogenous. Fabrics were dried for three nights, then labelled and imaged prior to washing. Two stains were added to 45 g knitted cotton ballast with 10 g SBL soiled ballast sheets (WFK, SBL2004.131‐930) and washed in a 1 L wash volume using an automated tergotometer (Peerless Systems, Washington, UK) at 35°C for 40 min, 300 rpm pot agitation in 19 US gpg hardness, then rinsed for 5 min twice at 15°C with an agitation of 208 rpm. Tide India heavy duty granular (HDG) laundry detergent (nil bleach, pH 10.5, Batch FP‐21‐002616/15) was used as the base detergent at recommended dose (3.75 g/L), with or without 2 PPM *Alkalitalea* GH16 enzyme as indicated. Two internal replicates and four external repeats were taken per treatment. Error bars displayed as standard error.

For mannan‐containing stain removal assessment, two replicates of 5 cm^2^ from each of Balsamic Salad Dressing (CFT, KC‐S‐406, Batch 028), Chocolate Ice Cream (2M Equest, M02 on EQWKC, Batch 48342), Chocolate Pudding (CFT, KC‐S‐69, Batch 022) and Guar Gum with Carbon Black (CFT, KC‐S‐43, Batch 073) stains were added to 40 g knitted cotton ballast with 10 g SBL soiled ballast sheets (WFK, SBL2004) and washed in a 1 L wash volume using an automated tergotometer (Peerless Systems, Washington, UK) at 35°C for 40 min, 300 rpm pot agitation in 19 US gpg hardness. These were then rinsed for 5 min twice at 15°C with an agitation of 208 rpm. Either tide India heavy‐duty granular (HDG) (nil bleach, pH 10.5, Batch FP‐21‐002616/15) or UK Fairy non bio heavy‐duty liquid (HDL) laundry detergent (batch code: 5098030350 02:50, pH 8) was used as the base detergent at recommended dose (3.75 g/L HDG, 2.4 mL/L HDL), with or without 2 PPM *Alkalitalea* GH26 enzyme as indicated. Two internal replicates and four external repeats were taken per treatment. Error bars displayed as standard error.

Stain removal was calculated by measuring L*a*b* values of washed stain swatches, unwashed stain swatches and unsoiled fabric, using a ColourEye 7000A Solid State Spectrophotometer (X‐Rite Europe GmbH) (Neiditch et al. 1980; Yau et al. 2024). Delta E* was then calculated by determining the staining intensity for the washed and unwashed stains compared to the unsoiled fabric, using the following equation where the suffix 1 denotes the unsoiled fabric value and the suffix 2 denotes the values for the unwashed or washed stains:

$$\Delta \;E_{\mathrm{ab}}^{\cdot }=\sqrt{{\left(L_{2}^{\cdot }-L_{1}^{\cdot }\right)}^{2}+{\left(a_{2}^{\cdot }-a_{1}^{\cdot }\right)}^{2}+{\left(b_{2}^{\cdot }-b_{1}^{\cdot }\right)}^{2}}$$

The Stain Removal Index (SRI) is the level of stain removal calculated as a percentage as follows: SRI = 100 × (*A*−*B*)/*A*; where *A* represents ΔE* of unwashed fabric‐stained region and *B* represents ΔE* of washed fabric‐stained region.

## 3 Results

### 3.1 Bacterial Growth on Different Carbon Sources

**FIGURE 1:**
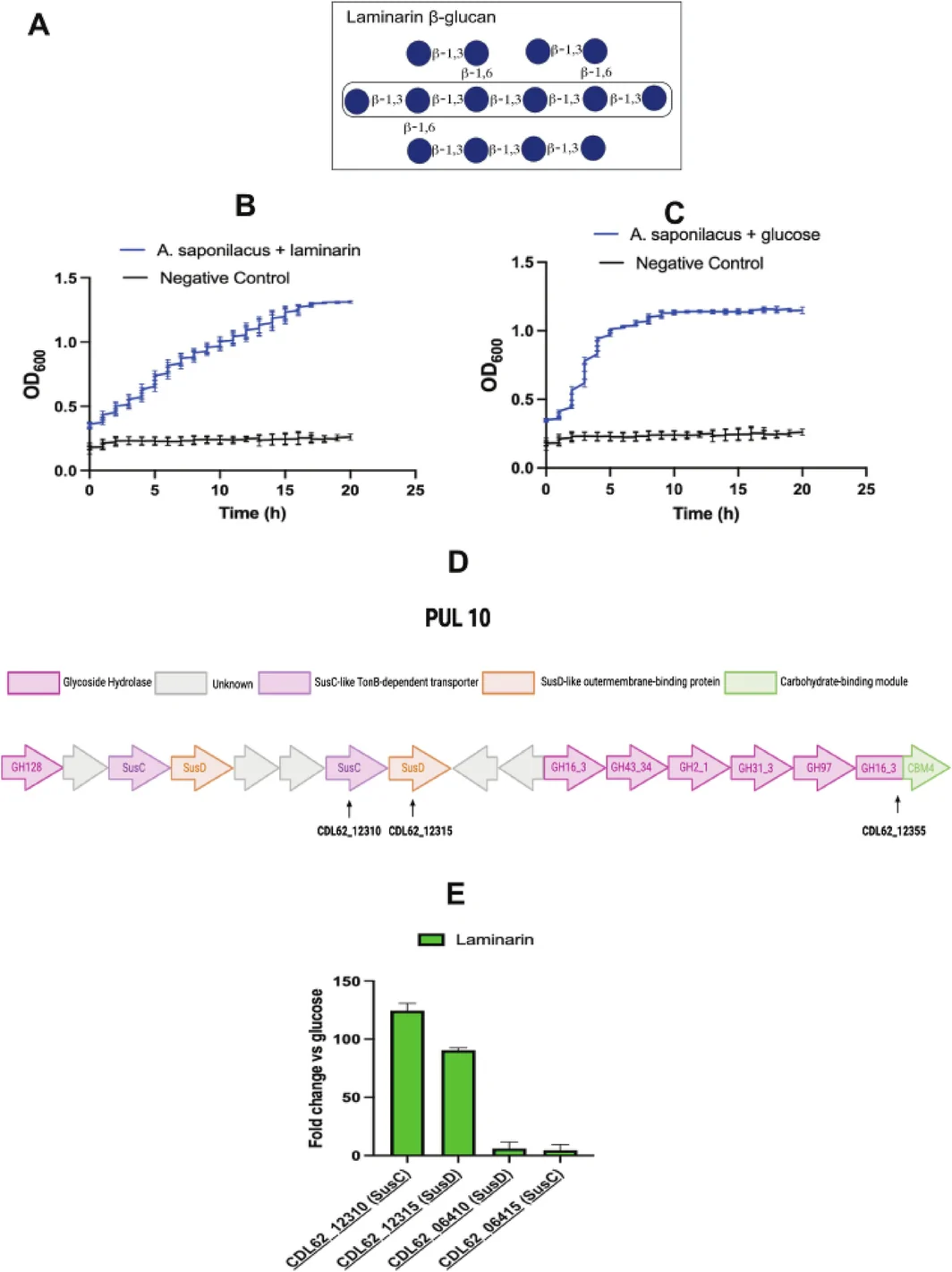
(A) Structure of laminarin. (B, C) *A. saponilacus* growth on laminarin and glucose, respectively. Negative control was performed with minimal media and carbon source alone, without *A. saponilacus*. (D) PUL composition of *A. saponilacus* PUL 10 (predicted as β‐glucan‐active). (E) RT‐qPCR on SuS‐C/SusD‐like genes.

To evaluate the potential of *Alkalitalea saponilacus* as a foundational organism for alkaline‐based biotechnological applications, namely high pH (9.5–11.0) detergent formulations, its ability to utilise various mono‐ and polysaccharides as sole carbon sources was assessed. *A. saponilacus* exhibited growth primarily on beta‐glucan and xylan‐based substrates. Highest final OD_600_ was observed when birchwood glucuronoxylan (1.41), tamarind xyloglucan (1.32) laminarin (β‐1,3‐1,6‐glucan) (1.45) and barley β‐glucan (β‐1,3‐1,4‐glucan) (1.02) were supplied, demonstrating the ability of this bacterium to degrade these complex polysaccharides (Figure S1A,B and Figure 1A–C, Table S2). A discernible lack of growth on other complex polysaccharides, including pectin (polygalacturonic acid) and gum arabic (arabinogalactan protein) suggests a lack of the necessary enzymes and/or transporters to fully degrade or import these substrates (Table S2) (McKee et al. 2021).

In addition to complex glycans, the ability of *A. saponilacus* to use only monosaccharides present in their respective polymers was also explored; this included glucose, galactose, xylose, arabinose, glucuronic acid and galacturonic acid. Figure 1C and Figure S1C show the ability of *A. saponilacus* to utilise glucose and xylose to support growth. This observed monosaccharide metabolism supports polysaccharide growth trends seen in *A. saponilacus*, showing a clear preference for xylose and glucose‐based carbohydrates.

### 3.2 Predicted PUL Architecture

The marked preference of *A. saponilacus* for β‐glucan based substrates, warranted a further deep‐dive into the bacterial degradation of these saccharides. To do this, polysaccharide utilisation loci (PULs), as the primary genetic systems underpinning polysaccharide breakdown, were selected as the focal point of this work. *A. saponilacus* encodes 14 predicted PULs of which only PUL 5, 8, 10 and 14 indicate potential β‐glucan specificity due to the encoded enzyme classes (Terrapon et al. 2018). PUL 10 was selected for further investigation due to both the increased number of predicted enzymes and the inclusion of glycoside hydrolase family 16, 128 and carbohydrate binding module 4 within this PUL (Drula et al. 2022). Xylan‐predicted PUL 7 was also examined for comparison due to both *A. saponilacus* growth on xylan‐based substrates and xylanase activity having been previously reported in *A. saponilacus* (Liao et al. 2018).

Figure 1D shows the genetic organisation of an *A. saponilacus* PUL 10, a putative cluster involved in the metabolism of β‐glucan substrates. This PUL encodes a GH128 protein (predicted endo‐ or exo‐β‐1,3‐glucosidase, CDL62_12280), a GH43_34 (predicted α‐L‐arabinofuranosidase, CDL62_12335), a GH2_1 (predicted β‐galactosidase, CDL62_12340), a GH31_3 (predicted α‐xylosidase, CDL62_12345), a GH97 (α‐galactosidase, CDL62_12350) and two classical GH16_3 (predicted to be active as endo‐glucanases on laminarin and/or barley β‐glucan, CDL62_12355, CDL62_12330). As PUL 10 is predicted to encode enzymes targeting more than glucose‐based glycans alone, it is anticipated that this PUL targets complex β‐glucan‐based substrates from various sources, containing multiple side chain variants. This could include xyloglucan, where xylose and glucose have been reported to be linked through an α‐linkage (Larsbrink et al. 2014). Figure S1 shows the genetic organisation of an *A. saponilacus* PUL 7, predicted to be utilised for the breakdown of xylan‐containing substrates. This PUL contains a GH43_12 containing a CBM91 (predicted β‐1,4‐xylosidase/α‐L‐arabinofuranosidase CDL62_06380), a GH10 (CDL62_06400), and a GH11 (CDL62_06390), both classically described in xylan metabolism; and a GH5_21 (CDL62_06395), annotated as an endo‐β‐1,4‐xylosidase. Overall, this PUL presents as a classical xylan PUL involved in the metabolism of various xylan‐based structures.

### 3.3 RT‐qPCR on Selected Genes

In order to confirm the involvement of PUL 10 β‐glucan metabolism, RT‐qPCR was performed on two selected genes within this PUL. SusC/SusD pairs were chosen due to their strong up‐regulation in response to the cognate polysaccharide in the described Sus‐like system (Foley et al. 2016). Specific primers were designed for CDL62_12310 (SusC) and CDL62_12315 (SusD). As a specificity comparison, the equivalent SusC/SusD pair from PUL 7 (CDL62_06410, CDL62_06415) was tested under the same conditions. PUL 7 gene expression was quantified in response to *A. saponilacus* growth with xylan compared to xylose, whereas PUL 10 gene expression was quantified in response to growth with laminarin (β‐1,3/1,6‐glucan) compared to glucose (Figure 1E).

The marked upregulation of the selected SusC/D pairs in PUL 7 when *A. saponilacus* was grown with xylan is consistent with a predicted role of this PUL in xylan metabolism. Similarly, PUL 10 can be confirmed as a locus associated with the degradation of long chain β‐glucan, due to gene upregulation under laminarin presence (Figure S1E). The fact that PUL 10 SusC/D expression was strongly induced by laminarin but not xylan, while PUL 7 SusC/D expression was induced by xylan but not laminarin, confirms that PUL 10 induction reflects specific β‐glucan responsiveness rather than a general response to complex polysaccharides. Together, these results confirm PUL 10 as the locus specifically associated with long‐chain β‐glucan degradation in *A. saponilacus*.

### 3.4 Secretome Prediction Based on Detergent Relevant Enzyme Classes

Given the highly alkaline conditions of heavy‐duty granular and automatic dishwashing detergents and the presence of β‐glucans in consumer‐relevant stains, the confirmation of *A. saponilacus* as an alkaline‐stable β‐glucan degrader motivated further proteomic investigation for this application.

The UniProt proteome UP000191055 from *Alkalitalea saponilacus* DSM 24412 was used as a query and facilitated the identification of 3564 encoded proteins both secreted and cellularly internalised (UniProt 2025). These were manually reviewed and reduced to 190 candidates based on a predicted enzyme activity (Dalkiran et al. 2018). Whilst glycoside hydrolases were a focal point of this selection, other detergent relevant enzyme classes were considered including polysaccharide lyases, lipases/esterases, nucleases and proteases (Table S3). A further filtration step prioritised proteins predicted to be secreted extracellularly, as these enzymes would be directly exposed to the highly alkaline, carbonate‐rich environment of the soda lake and would therefore be expected to possess adaptations necessary for extracellular activity (Savojardo et al. 2018).

Endo‐acting enzymes were also selected, as they are typically more effective at breaking down the large polymeric substrates targeted by detergent enzymes than that of exo‐acting enzymes. Enzymes greater than 100 kDa were excluded in initial screening, as high molecular‐weight proteins are more difficult to express and produce at a scale necessary for the desired industrial application. These selection criteria produced a final shortlist of 54 candidates for heterologous production (Table S3).

### 3.5 Expression and Initial Characterisation of Selected *A. saponilacus* CAZymes

**FIGURE 2:**
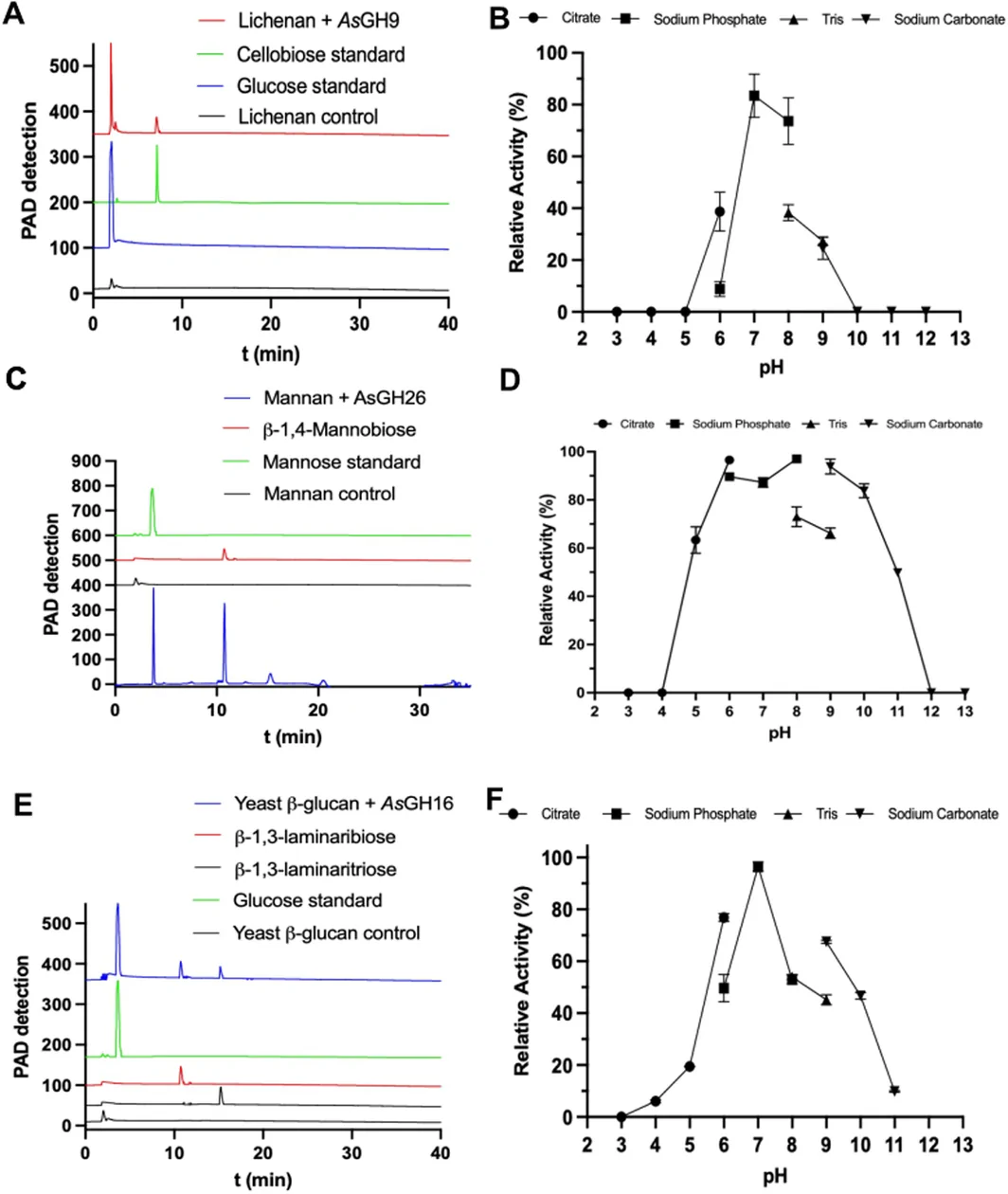
(A) HPAEC of *As*GH9 with lichenan (β‐1,3/1,4‐glucan). (B) HPAEC of *As*GH26 with ivory nut mannan (β‐1,4‐mannan). (C) HPAEC of *As*GH16 with yeast β‐glucan (β‐1,3/1,6‐glucan). (D) pH profile of *As*GH9 with lichenan (β‐1,3/1,4‐glucan). (E) pH profile of *As*GH26 with glucomannan (β‐1,4‐glucomannan) (F) pH profile of *As*GH16 with curdlan (β‐1,3‐glucan).

As shown in Table S3, *A. saponilacus* encodes a variety of secreted carbohydrate active enzymes with predicted detergent‐relevant activities. To validate the ability of these proteins to perform under harsh alkaline conditions and degrade relevant stain components, three glycoside hydrolases (*As*GH9, *As*GH16 and *As*GH26) were heterologously expressed. As PUL 10 was experimentally confirmed as β‐glucan degrading, the GHs encoded in this PUL represent a promising starting point for proof‐of‐concept enzyme selection. For this reason, in addition to carbohydrate‐binding module presence, facilitating insoluble substrate degradation, GH16_3 (AsGH16) (CDL62_13555) was selected for production (Figure 1E). To support the value of *A. saponilacus* enzymes, across GH families and PUL involvement, a GH9 (AsGH9) (CDL62_13645) present in PUL 13 and a GH26 (AsGH26) (CDL62_12545) not present in a PUL were also expressed. Following expression in *E. coli* BL21 (DE3), the activity of AsGH16, AsGH26 and AsGH9 was characterised via TLC (Figure S2) and HPAEC (Figure 2A,C,E).

AsGH16 was confirmed as an endo‐β‐1,3‐glucanase with primary activity on linear β‐1,3‐glucan, paramylon, and secondary activity on branched β‐1,3/1,6‐glucan derived from yeast. *As*GH16 cleaved both paramylon and yeast β‐glucan into laminaritriose (β‐D‐1,3‐glucotriose), laminaribiose (3‐β‐D‐Glucopyranosyl‐(1 → 3)‐D‐glucose), and glucose but showed no activity on pustulan (Linear β‐1,6‐glucan) or β‐1,3‐glucooligosaccharides (Figure 2C). It is likely that *As*GH16 has an affinity for the β‐1,3‐linked glucose backbones of these substrates, and activity on yeast β‐1,3/1,6‐glucan is slightly reduced due to steric hindrance caused by the β‐1,6‐glucose branches. This is reaffirmed by the lack of activity of AsGH16 on β‐1,6‐glucan alone, suggesting AsGH16 does not degrade this bond type. Although the optimum pH of *As*GH16 was found to be pH 7 (Figure 2F), a retained 60% relative activity at pH 9–10, rather than an immediate drop‐off, can be observed. This trend is established across many extreme‐adapted enzymes and can represent a bacterial advantage towards retaining neutral pH activity, likely reflecting selective pressure to preserve catalytic efficiency under the transient climate of Soap Lake (Dhakar and Pandey 2016; Li et al. 2020).

AsGH26 was confirmed as an endo‐β‐1,4‐mannanase active on ivory nut mannan, carob galactomannan and glucomannan. In a similar manner to *As*GH16, branching appeared to hinder catalytic activity in *As*GH26, again suggesting either a preference for linear substrates or a favouring of β‐1,4 bond types. The extended‐cleft shaped active sites of endo‐acting enzymes often favour structural interactions with linear substrates, perhaps explaining the steric hinderance experienced when branched substrates are introduced (Morais et al. 2023). Similarly observed with AsGH16, a broadened pH range rather than a shift in optimum pH can be seen in AsGH26, with 83.8% of enzyme activity retained at pH 10 (Figure 2D).

Glycoside Hydrolase family 9 has been previously documented as active on xyloglucan, xanthan, cellulose, mixed linkage β‐glucan and even birchwood xylan (Mandelman et al. 2003; Moroz et al. 2018; Foley et al. 2019). In the case of *As*GH9, activity was detected on β‐1,3/1,4‐glucan releasing glucose and glucobiose as unique final products and on xyloglucan, releasing a large oligosaccharide (Figure 2A, Figure S1A). The similar β‐1,4‐glucose linkages in both lichenan and xyloglucan backbones, could be the prime recognition site of *As*GH9, however the lack of activity on carboxymethyl cellulose (CMC) challenges this interpretation. It is possible that carboxymethyl substitutions prevent enzyme/polysaccharide recognition, though *As*GH9 appears capable of binding branched xyloglucan. In contrast to AsGH16 and AsGH26, AsGH9 displays a pH profile more typical of a neutral adapted enzyme (Figure 2B).

### 3.6 Structural Characterisation of Selected *A. saponilacus* CAZymes

The three‐dimensional structures of expressed *A. saponilacus* enzymes were predicted using AlphaFold (Figure S3). Figure S3A shows the overall predicted structure of *As*GH16. The N‐terminal catalytic domain (~29.86 kDa) is attached to three carbohydrate binding modules, one annotated as a CBM 4 and two tandem CBM 30s (approximately 18.05 kDa, 16.82 kDa, 16.72 kDa, respectively). A small Por secretion system C‐terminal sorting domain is connected to these non‐catalytic binding domains, further suggesting that *As*GH16 likely functions either at the cell surface or extracellularly (Mizgalska et al. 2024). The catalytic residues seen in *As*GH16 are highly conserved when compared with canonical non‐alkaline GH16s described in literature. As seen in Figure S3A, E143 and E148 are perfectly positioned to act as the catalytic nucleophile and acid/base and the conserved GH16 EXDXXE motif was identified (Planas 2000). The residue M147 lies within this region and is associated with the characteristic β‐bulge, a feature that distinguishes AsGH16 from canonical bacterial mixed‐linkage glucanases, which instead possess a regular β‐strand lacking this insertion (Viborg et al. 2019).

Structural models were also produced for *As*GH9 and *As*GH26 (Figure S3B,C, respectively). *As*GH26 showed a classical (β/α)_8_ barrel with residues E192 and E299 acting as the catalytic acid/base and nucleophile, respectively. *As*GH9 was predicted to have a standard (α/α)_6_ barrel architecture, with D185 and E550 functioning as the catalytic base and acid, respectively (Figure S3B). In contrast to *As*GH16, no CBMs were observed in either *As*GH26 (Figure S3C) or *As*GH9, although a small (8.49 kDa) immunoglobulin‐like domain was present at the N‐terminal of AsGH9 (Figure S3B). The insolubility of *As*GH9 upon removal of this immunoglobulin‐like domain suggests that the domain plays a key role in facilitating soluble folding of the protein.

### 3.7 Alkaline‐Typical Features of Selected *A. saponilacus* CAZymes

As is widely documented in enzymes from extreme environments, all three *A. saponilacus* enzymes displayed an increased number of negatively charged residues on the protein surface (Karan et al. 2020). Regional charge distribution is shown in Figure S4, whilst the net charge of both folded and unfolded enzymes is shown in Tables S4–S6. *As*GH9 (Figure S4C) and *As*GH26 (Figure S4B) maintained a folded net charge of −7.46 and −8.71 per 100 amino acids at pH 10, whereas *As*GH16 (Figure S4A) was more strongly ionised at this pH, with a net charge of −10.98 per 100 amino acids (Tables S4–S6).

To characterise the structural stability of *As*GH16, *As*GH26 and *As*GH9, four features, characteristic of extreme‐adapted enzymes were assessed; lysine and arginine content, hydrogen bonding network extent, hydrophobic core packing, and disulfide bond content (Table 1) (Zhao et al. 2011; Suplatov et al. 2014; Sáez‐Jiménez et al. 2015). Across all three enzymes lysine content was notably reduced relative to typical protein composition, ranging from 1.7% (*As*GH16) to 4.7% (*As*GH9) with *As*GH26 as an intermediate (3.6%) (Bairoch 2000). This reduction, however, does not seem to have been compensated with an increase in arginine content, which ranged from 3.1% (*As*GH9) to 5% (*As*GH26), with *As*GH9 representing the only candidate where lysine outnumbered arginine (Lys:Arg = 1.53). This may reflect the neutral adapted traits of *As*GH9. *As*GH26 showed the highest hydrogen bond density (104.7 per 100 residues), whilst *As*GH16 showed the lowest hydrogen bond density (79.9 per 100 residues). All enzymes demonstrated a well packed hydrophobic core with *As*GH26 demonstrating the greatest hydrophobic burial (90.29% buried SASA) and the smallest internal cavity volume (129.38 ų). *As*GH9, by contrast, showed intermediate hydrophobic burial (87.55%) but by far the largest cavity volume (800.53 ų) of the three, indicating that its hydrophobic residues, while reasonably well shielded from solvent, are packed with considerably more internal void space than either *As*GH16 (284.3 ų) or *As*GH26. Disulfide bridge content was low across all enzymes with only *As*GH9 containing a disulfide bond.

**TABLE 1:** Structural and compositional features of three candidate alkaline enzymes (*As*GH16, *As*GH26, *As*GH9).

Metric	*As*GH16	*As*GH26	*As*GH9
Lys residues (*n*)	15	13	26
Lys content (%)	1.7	3.6	4.7
Arg residues (*n*)	29	18	17
Arg content (%)	3.3	5.0	3.1
H‐bonds (total)	697	380	510
H‐bonds (backbone only)	566	247	366
H‐bonds per 100 aa	79.9	104.7	92.1
Buried hydrophobic SASA (%)	82.53	90.29	87.55
Internal cavity volume (Å^3^)	284.3	129.38	800.53
Disulfide bonds (*n*)	0	0	1
Disulfides per 100 aa	0	0	0.2

*Note:* Lysine and arginine content are reported for the mature (signal‐peptide‐removed) sequence. Hydrogen bonds are reported as backbone‐only (DSSP) and total, all‐atom (MDAnalysis), normalised per 100 residues. Buried hydrophobic SASA reflects the mean percentage burial of hydrophobic residues (Ala, Val, Leu, Ile, Phe, Met, Trp). Internal cavity volume excludes pockets corresponding to catalytic active sites, interdomain interfaces, or low‐confidence structural region.

### 3.8 Detergent‐Relevant Performance of Alkaline Glycoside Hydrolases

To assess the prospects of *As*GH9, *As*GH16 and *As*GH26 as detergent additives, the respective molecules were screened for performance benefits across various through‐the‐wash assessment vectors. *As*GH16 and *As*GH26 were screened for stain removal against β‐glucan and mannan containing stains, respectively, whilst *As*GH9 was screened for the ability to minimise whiteness loss associated with soil redeposition via fabric cellulose degradation (Calvimontes et al. 2011, 2012; Yau et al. 2024).

**FIGURE 3:**
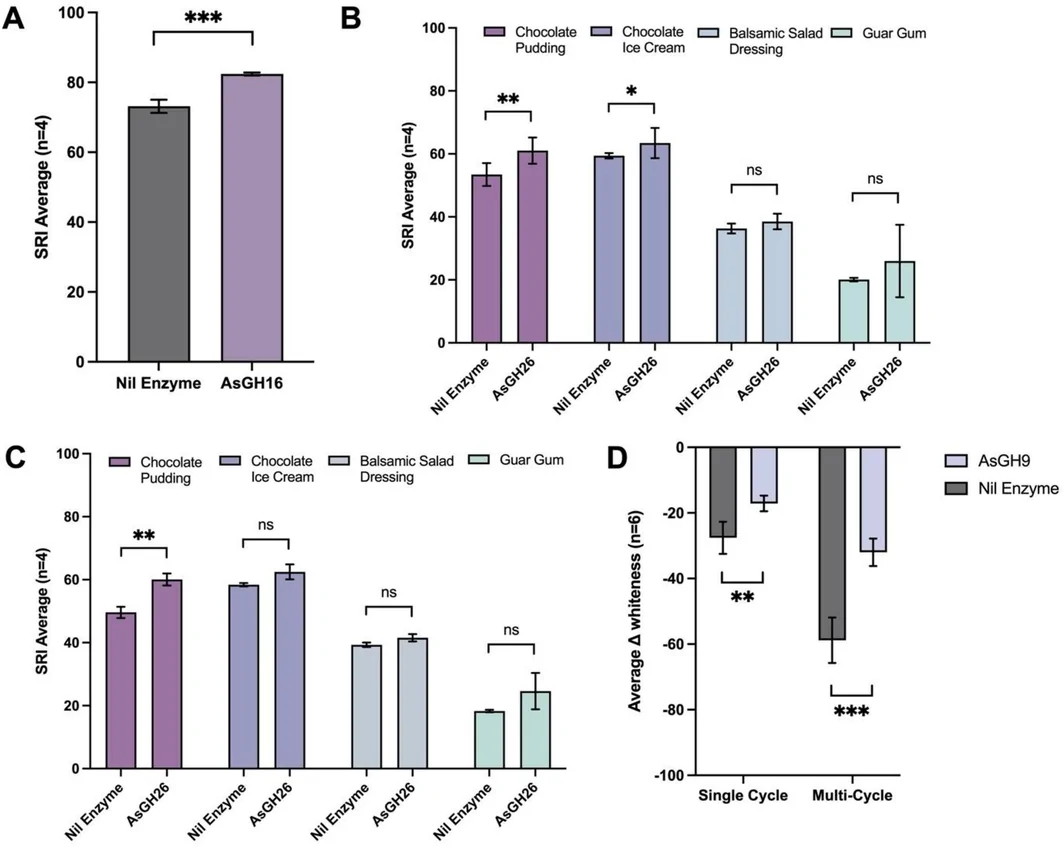
(A) Make‐up stain removal performance of *As*GH16. (B) Stain removal performance of *As*GH26 in heavy‐duty granular (HDG) detergent (pH 10.5). (C) Stain removal performance of *As*GH26 in heavy‐duty liquid (HDL) detergent (pH 8). (D) Single and multi‐cycle whiteness performance of *As*GH9. All assays compared the respective enzyme treatment vs. nil enzyme. Statistical significance was determined using a Student's *t*‐test, with significance indicated using the standard asterisk convention (*p < 0.05, **p < 0.01, *** *p* < 0.001; ns, not significant).

This screening found the addition of all *A. saponilacus* derived enzymes (AsGH16, AsGH26 and AsGH9) into current detergent formulas to provide quantifiable cleaning improvement (Figure 3). The addition of *As*GH16 to detergent delivered a statistically significant (10%) improvement on make‐up stain removal compared to that of the nil enzyme control (Figure 3A). *As*GH26 was found to improve removal of various mannan‐containing food stains (chocolate pudding, chocolate ice cream, balsamic salad dressing) and fabric stained with guar gum in both HDL (pH 8) and HDG detergents (pH 10.5), though significance was only noted in that of chocolate ice cream (HDG) and chocolate pudding (HDL, HDG) (Figure 3B,C respectively). The activity of *As*GH26 both in HDG and HDL detergents is consistent with the broad pH range seen in Figure 2D and represents strong industrial prospects for this enzyme.

Knitted cotton fabric and clean cotton ballast fabric were washed in the presence of particulate soil and Fairy HDL detergent either with or without *As*GH9. Change in textile whiteness index (ΔWI) after one (single) or four (multi) wash cycles in the presence of particulate soil was calculated as whiteness loss; thus, a less negative ΔWI value defines an improvement in whiteness. The addition of *As*GH9 into detergent resulted in both visible and quantifiable fabric whiteness improvement (Figure 3D). Multi‐cycle washing with *As*GH9 spiked into HDL detergent produced fabrics with a whiteness index of −32.0 ± 4.2 whiteness units, whilst washing in the absence of *As*GH9 produced an average whiteness index of −58.8 ± 6.9 whiteness units.

In the case of all enzymes, *As*GH16, *As*GH26 and *As*GH9, activity could be seen in the presence of surfactants, builders and the high carbonate concentrations typical of commercial detergent formulations. These results clearly demonstrate the compatibility of *A. saponilacus* with current detergent formulations and reinforce the strong prospects of these candidates in industry.

## 4 Discussion

Enzymes have been established as a key technology in driving the decarbonisation of industrial processes and increasing their sustainability (Radley et al. 2023). The use of enzymes in detergents has revolutionised the field, reducing the dependence on high washing temperatures and long cycle lengths, thereby lowering fossil fuel use (Molina‐Espeja et al. 2023). Despite this, the extreme alkalinity of detergent matrices has limited the activity of enzymes in these formulations as most are typically isolated from mesophilic bacteria (Espina et al. 2021). *Alkalitalea saponilacus* is an obligate anaerobe that thrives under the alkalinity and moderate salinity of Soap Lake and has been shown to use a range of mono‐ and polysaccharides as carbon sources. However, only one study to date has characterised the enzymology of this bacterium, despite its clear potential across a range of biotechnological applications (Liao et al. 2018). Liao et al. described the ability of *A. saponilacus* to use xylan as a carbon source, and bioinformatically identified candidate metabolic pathways, proposing the secretion of a GH10 enzyme as a likely response mechanism.

In this study, we systematically characterised the metabolic versatility of *A. saponilacus* with respect to its potential industrial application, focusing on detergent use. We confirmed the bacterium's capacity to utilise diverse carbon sources such as laminarin, glucuronoxylan and mixed‐linkage barley β‐glucan (Figure 1 and Figure S1; Table S2). *A. saponilacus* was also able to metabolise other glycans, albeit to a lesser extent, as evidenced by the comparatively lower growth on these polysaccharides. Specifically, the bacterium demonstrated growth on carboxymethyl cellulose (CMC), birchwood glucuronoxylan, wheat arabinoxylan and alginate, achieving final ODs ranging from 0.2 to 0.6 (Table S2). This reduced growth may be attributable to the absence of key enzymes required for the initial depolymerization at the bacterial cell surface or a lack of necessary enzymes in the periplasm to fully degrade these polymers (Grondin et al. 2017). The preference of *A. saponilacus* for monosaccharides such as glucose, galactose and xylose supports the efforts of this bacterium to degrade complex sugars composed of these units.

Following the selection of *A. saponilacus* beta‐glucan metabolism as a focal point, PUL 10 was confirmed as laminarin degrading using RT‐qPCR with the SusC/SusD pair (Figure 2). This PUL was predicted to be more complex than other potential β‐glucan degrading PULs in *A. saponilacus* and encodes a GH128, two GH16_3, a GH43_34, a GH2_1, a GH31_3 and a GH97. This combination of enzymes is canonical of bacterial PULs, in which endo‐acting enzymes cleave a polymer backbone into oligosaccharides at the cell surface, while exo‐acting glycosidases subsequently trim these oligosaccharides into monosaccharides suitable for periplasmic or cytoplasmic uptake and fermentation (Foley et al. 2016). The duplication of GH16_3s in PUL 10 is notable as this enzyme class is consistently associated with laminarin and mixed‐linkage glucan degradation across both marine and gut‐associated Bacteroidetes. They form the catalytic core of laminarin utilisation loci in human gut *Bacteroides* species as well as marine species *Zobellia galactanivorans* , where they are frequently paired with a laminarin‐binding carbohydrate‐binding module (CBM) and surface glycan‐binding protein (Labourel et al. 2015; Déjean et al. 2020; Zühlke et al. 2024). The presence of two GH16s within this PUL may represent either functional redundancy or different substrate specificity in terms of linkage preference. The remaining enzymes within PUL 10 are consistent with a debranching role, potentially removing side‐chain decorations from the substrate prior to backbone hydrolysis by endo‐acting GH16 and GH128 (Déjean et al. 2020). The fact that GH97 and GH31 are alpha‐acting enzymes suggests that either the overall substrate of PUL 10 contains a degree of alpha‐linked side chain decoration or that these enzymes act on a co‐occurring alpha‐glucan/galactan present in algal material *A. saponilacus* encounters within the sediments of Soap Lake (Naumoff 2005; Arumapperuma et al. 2023).

Following the experimental validation of PUL 10 as involved in *A. saponilacus* β‐glucan degradation, the published proteome of this bacterium was analysed to identify candidates predicted to be extracellularly secreted. *A. saponilacus* is known to employ a ‘compatible‐solute strategy’ to maintain cytoplasmic pH homeostasis under conditions of high salinity and alkalinity (Liao et al. 2018). Accordingly, to identify enzymes adapted to function at an alkaline pH, we focused on those predicted to be secreted from the cell (Table S3). The initial focal point of this analysis was beta‐glucan degrading enzymes, largely due to the lack of alkaline non‐starch polysaccharide degraders in commercial detergents, however candidate identification was also broadened to other detergent‐relevant enzyme classes.

The identification of over 50 secreted enzymes highlights *A. saponilacus* as a promising source of naturally alkaline‐stable enzymes, with potential application in detergent optimisation. Whilst secondary to mesophilic candidates, some examples of alkaline bacteria have been previously exploited as sources of alkaline CAZymes for detergent formulations (Dhundale et al. 2026). Strains such as *Bacillus* KSM‐19, KSM‐64 and KSM‐520 have been reported to produce cellulases effective at enhancing cleaning efficiency within detergent formulas; however, these bacteria are predominantly limited to cellulase production and exhibit strong activity mainly against carboxymethyl cellulose (Shikata et al. 1990; Dhundale et al. 2026). In contrast, *A. saponilacus* demonstrates considerable metabolic versatility, both in its capability of utilising multiple complex carbon sources and its plethora of extracellularly secreted enzymes. This underscores the bacterium's potential, not only within the detergent industry but also for broader biotechnological uses, such as the processing of cellulose and xylans relevant to the paper and biomass industries.

As a proof of concept, we selected three enzymes from the *A. saponilacus* secretome: *As*GH9 (CDL62_13645), *As*GH16 (CDL62_12355) and *As*GH26 (CDL62_12545), which are predicted to function as endo‐glucanases (*As*GH9 and *As*GH16) and an endo‐mannanase (*As*GH26). These enzymes were selected for various industrial and investigative reasons. Firstly, the selection of *As*GH16, a candidate within β‐glucan PUL 10, *As*GH9 within PUL 13 and *As*GH26, not associated with a PUL, was designed to evaluate *A. saponilacus*' potential across varying genomic locations. Additionally, the predicted targets of these candidates (mannans and beta‐glucans) are highly relevant in current consumer detergent failures, both in fabric and dish soils, highlighting a ubiquitous unmet need (Runavot et al. 2014; Bakshani et al. 2023). The predicted endo‐acting capabilities of *As*GH16, *As*GH26 and *As*GH9 were also relevant selection criteria, as the degradation of complex polysaccharides into soluble oligosaccharides is an enzymatic feature commonly harnessed in detergent formulations to lift stain components into solution.

Recombinant soluble expression of these enzymes was successfully achieved in *Escherichia coli* BL21(DE3), and in vitro activity assays were conducted using a range of polysaccharide substrates (Figure S2 for TLC and Figure 2 for HPAEC). The confirmation of all three enzymes as both active and endo‐acting reaffirmed the industrial value of such candidates (Figure 2A,C,E). *As*GH9 exhibited activity towards lichenan (mixed‐linkage 1,3;1,4‐β‐glucan) and tamarind xyloglucan; *As*GH16 was active on paramylon and yeast β‐glucan (1,3;1,6‐β‐glucan); and *As*GH26 exhibited activity on various mannan types. While these activities are consistent with those previously reported for their respective glycoside hydrolase families, the stability of these enzymes at high pH, carbonate and sulfide concentrations highlights their potential suitability as detergent ingredients (Asao et al. 2011).

The pH trends seen in *As*GH16 and *As*GH26 activity, whilst slightly differing, show a similar overarching trend of a neutral optimum pH with retained alkaline activity, rather than a shifted optimum pH. This breadth of functional range is a hallmark of enzymes from extremophilic organisms which may encounter fluctuating environmental pH, particularly at the sediment–water interface or near microbial mats, where fermentation products and microbial activity can alter pH (Haines et al. 2023). Given the extreme conditions of Soap Lake, it is possible that *As*GH16 and *As*GH26 operate under a slightly reduced rate at higher pH due to a greater selective pressure to maintain structural stability than to maximise catalytic rate, likely due to the comparatively low microbial competition in this environment (Stark et al. 2022). Interestingly, the notably higher alkaline activity of *A. saponilacus* candidates *As*GH16 and *As*GH26 in carbonate buffer, relative to Tris, may reflect structural stabilisation by carbonate ions, mirroring the ionic conditions of Soap Lake, their native environment. Whilst the evolutionary pattern of a broadened pH peak over a shifted optimum is microbially valuable, this also represents industrial benefit by positioning *A. saponilacus* candidates as functional across various pH detergents including both heavy‐duty liquid (pH 8) and heavy‐duty granular (pH 10.5). *As*GH9 displays a classic neutral optimised pH profile. Whilst opposing other studied *A. saponilacus* enzymes, this may reflect action within the wall‐associated microenvironment, retention of an ancestral active‐site residue framework under stronger selection for catalytic/structural fitness than pH tolerance, or environmental pH change, where enzyme expression responds to seasonal changes causing reduced pH.

Structural predictions generated by AlphaFold3 revealed an increased prevalence of negatively charged residues on the solvent accessible surfaces, alongside net negative charges at both neutral and alkaline pH across all three enzymes (Figure S3, Tables S3–S5). This conserved surface electronegativity likely represents a molecular adaptation conferring stability under extreme environmental alkaline conditions (Zhao et al. 2011). The catalytic residues identified in *As*GH16, *As*GH26 and *As*GH9, a glutamate pair in *As*GH16 and *As*GH26, and an aspartate–glutamate pair in *As*GH9, reflect the reliance of these enzymes on complementary protonation states to maintain catalysis. At higher pH, where both residues are heavily deprotonated, the active site microenvironment is required to shift each residue's effective pKa away from its free amino acid value, typically through partial burial, electrostatic interaction between the two nearby carboxylates, and local hydrogen bonding (Wan et al. 2015). Although alkaline catalysis is valuable in these enzymes, alkaline stability and the ability to remain folded in such extreme conditions is arguably of greater importance.

The structural features typically associated with extreme environment tolerance, namely Lys:Arg content, hydrogen bonding, hydrophobic core packing and disulfide bonding, were evaluated in *As*GH16, *As*GH26 and *As*GH9 (Table 1). These features point to partially distinct stabilisation strategies across the three enzymes rather than a single alkaline adaptation mechanism. *As*GH26 relies largely on tight hydrophobic core packing and high hydrogen bond density to maintain non‐covalent structural reinforcement within this environment. However, *As*GH16 displays the most prominent lysine depletion yet lower hydrogen bonding, implying that electrostatic strategies and core structural reinforcement are somewhat independent alkaline stability features, rather than necessarily cooccurring. *As*GH9 shows arguably the weakest structural stability with intermediate hydrogen bonding, a less packed hydrophobic core and a weak Lys:Arg ratio. As it is the only enzyme to contain a disulfide bond this may partially offset the destabilising consequences of these features. Collectively, the structural features and catalytic residue arrangements observed across *As*GH16, *As*GH26 and *As*GH9 suggest these enzymes are structurally weighted towards stability rather than rapid catalytic turnover. This is a trait well suited both to the persistently alkaline, low‐competition conditions of Soap Lake and to detergent applications, where enzymes must remain functionally stable across extended wash cycles.

Finally, to confirm the ability of the representative *A. saponilacus* enzymes to deliver in‐wash cleaning benefits, they were screened as additives within current detergent formulas. Each enzyme delivered a statistically significant, quantifiable cleaning benefit consistent with its predicted substrate specificity. *As*GH16 improved make‐up stain removal relative to the nil‐enzyme control (Figure 3A), *As*GH26 delivered discernible mannan‐stain removal across both low and high pH detergent matrices (Figure 3B,C) and *As*GH9 reduced particulate soil redeposition, improving fabric whiteness across both single and multi‐cycle washing (Figure 3D). Beyond simply confirming activity, these results provide direct functional validation of several structural predictions. *As*GH26's effectiveness across both HDL and HDG formulations, which differ substantially in operating pH, is a direct applied demonstration of the broad pH‐activity plateau identified biochemically for this enzyme. Similarly, the persistence of *As*GH9's benefit across repeated wash cycles is consistent with a structural profile weighted towards stability over rapid catalytic turnover. This screening demonstrates not only that all three candidates are compatible with the surfactants, builders and carbonate concentrations typical of commercial formulations, but that the specific structural features distinguishing each enzyme directly underpin their differing, application relevant performance profiles.

In conclusion, this study validates *Alkalitalea saponilacus* as an excellent source of enzymes suited for biotechnological applications under highly alkaline conditions. The confirmation of *As*GH16, *As*GH26 and *As*GH9 as alkaline stable, detergent compatible candidates has been validated, not only structurally and biochemically, but also by their demonstrated activity within these matrices. The broad pH profiles observed across these enzymes represent a further advantage within the detergent industry, where pH varies across formulas, allowing for stain removal at both neutral and alkaline pH. Both *A. saponilacus* and its encoded enzymes warrant evaluation in other industrial processes, such as paper pulping, where alkaline pH environments are prevalent.

## Author Contributions

**A. Stephen:** conceptualisation, methodology, investigation, visualisation, writing – review and editing. **N. L. Brown:** methodology, investigation, visualisation. **R. Rostron:** investigation. **R. J. Wigham:** investigation, visualisation. **A. K. Malekpour:** investigation. **G. W. Black:** methodology, conceptualisation, writing – review and editing. **A. L. Morales Garcia:** conceptualisation, methodology, supervision, writing – review and editing. **H. C. L. Yau:** conceptualisation, methodology, supervision, writing – review and editing. **N. J. Lant:** methodology, project administration, writing – review and editing. **J. Munoz‐Munoz:** conceptualisation, methodology, investigation, visualisation, project administration, supervision, writing – original draft, writing – review and editing.

## Funding

This work was supported by the Biotechnology and Biological Sciences Research Council.

## Conflicts of Interest

H.C.L.Y., A.K.M., A.L.M.G., R.J.W. and N.J.L. are employed by Procter and Gamble Technical Centres Ltd., a subsidiary of the Procter & Gamble company, which has Intellectual Property interests relating to the application of materials disclosed in the article. H.C.L.Y, A.K.M., A.L.M.G., N.J.L., N.L.B., G.W.B. and J.M.‐M. are listed as inventors in this intellectual property. All other authors declare that they have no competing interest.

## Supporting information


**Table S1:** Primers used in this study.
**Table S2:** Carbon screening of *A. saponilacus* in minimal media using different sugars as sole carbon sources.
**Table S3:** Detergent‐relevant enzymes secreted by *A. saponilacus*.
**Table S4:** Net charge of AsGH16 across pH range. Values were calculated using PDB2PQR v3.7.1: biomolecular structure conversion software (Dolinsky et al. 2007; Olson et al. 20112011; Jurrus et al. 2018). Net charge is normalised to charge per 100 amino acids.
**Table S5:** Net charge of AsGH26 across pH range. Values were calculated using PDB2PQR v3.7.1: biomolecular structure conversion software (Dolinsky et al. 2007; Olson et al. 2011; Jurrus et al. 2018). Net charge is normalised to charge per 100 amino acids.
**Table S6:** Net charge of AsGH9 across pH range. Values were calculated using PDB2PQR v3.7.1: biomolecular structure conversion software (Dolinsky et al. 2007; Olson et al. 2011; Jurrus et al. 2018). Net charge is normalised to charge per 100 amino acids.
**Figure S1:** A. Structure of glucuroxylan. Growth curves on glucuroxylan (B) and xylose (C), respectively. Negative control was performed with minimal media and carbon source alone, without *A. saponilacus*. D. PUL composition of PUL (predicted as xylan‐active). E. RT‐qPCR on SuS‐C/SusD‐like genes.
**Figure S2:** (A) Activity of AsGH9 on potential substrates. (B) Activity of AsGH16 on potential substrates. (C) Activity of AsGH26 on potential substrates.
**Figure S3:** (A) Structure of *As*GH16 with catalytic residues shown in zoom. (B) Structure of *As*GH9 with catalytic residues shown in zoom. (C) Structure of *As*GH26 with catalytic residues shown in zoom.
**Figure S4:** (A) Structure of GH26 with surface charge. (B) GH26 surface charge. (C) Structure of GH9 with all CBMs. (D) GH9 surface charge.

## Data Availability

The data that support the findings of this study are available from the corresponding author upon reasonable request.
