# Supplementary material for: Exploration of Alkaliphilic Bacteria Alkalitalea saponilacus Identifies Glycoside Hydrolases With Biotechnological Potential
Source: Microb Biotechnol. 2026 Sep 21;19(9):e70448. doi: 10.1111/1751-7915.70448 (PMC13591233; doi:10.1111/1751-7915.70448)
Supplement: Supplementary file 1 [file MBT2-19-e70448-s001.docx]

**Supplementary Material**

**Table 1SM.** Primers used in this study.

| Gene | Primer Forward | Primer Reverse |
| --- | --- | --- |
| GH9: CDL62_13645 (A0A1T5F9N3) | NheI TGCCATAG GCT AGC TGC ACA GGA CAT GAA AAC A | XhoI TGCCATAG CTG GAG TTA GTT TTG TGT CAA CAA TGC GT |
| GH16: CDL62_12355 (A0A1T5CIE7) | NheI TGCCATAG GCT AGC CAG GAA TAC CAA CTT GTT T | XhoI TGCCATAG CTG GAG CTA TTT TTT AAT CAC AAT TCT |
| GH26: CDL62_12545 (A0A1T5CLR4) | NdeI TGCCATAG CAT ATG TGT AAC GAA ATC ACT GAA | XhoI TGCCATAG CTG GAG TTA TAA ACT ATT AAA CCT ATA |

**Table 2SM.** Carbon screening of *A. saponilacus* in minimal media using different sugars as sole carbon sources.

| Substrate | Final OD after 24 hours of growth |
| --- | --- |
| Polysaccharide: | |
| Laminarin | 1.45 |
| Barley β-glucan | 1.02 |
| Pectin | 0.05 |
| CMC | 0.10 |
| Birchwood glucuronoxylan | 1.41 |
| Wheat arabinoxylan | 0.11 |
| Tamarind xyloglucan | 1.32 |
| Gum Arabic | 0.03 |
| Alginate | 0.12 |
| Monosaccharide: | |
| Glucose | 1.18 |
| Galactose | 0.91 |
| Xylose | 1.22 |
| Arabinose | 0.42 |
| Glucuronic acid | 0.13 |
| Galacturonic acid | 0.04 |

**Table 3SM.** Detergent-relevant enzymes secreted by *A. saponilacus.*

| Entry | Entry Name | Protein names | Cellular location (BUSCA) | Location Prob | Protein families |
| --- | --- | --- | --- | --- | --- |
| A0A1T5CIE7 | A0A1T5CIE7_9BACT | Por secretion system C-terminal sorting domain-containing protein | extracellular space | 0.97 | Glycosyl hydrolase 16 family |
| A0A1T5HNV3 | A0A1T5HNV3_9BACT | Por secretion system C-terminal sorting domain-containing protein | extracellular space | 0.84 | Glycosyl hydrolase 16 family |
| A0A1T5CLR4 | A0A1T5CLR4_9BACT | Mannan endo-1,4-beta-mannosidase (EC 3.2.1.78) | extracellular space | 0.92 | Glycosyl hydrolase 26 family |
| A0A1T5A934 | A0A1T5A934_9BACT | Endo-1,4-beta-xylanase, GH35 family | cytoplasm | 0.7 | Glycosyl hydrolase 35 family |
| A0A1T5BSR4 | A0A1T5BSR4_9BACT | Endo-1,4-beta-xylanase (EC 3.2.1.8) | extracellular space | 0.97 | Glycosyl hydrolase 11 (cellulase G) family |
| A0A1T5CHB8 | A0A1T5CHB8_9BACT | Glycosyl hydrolases family 16 | plasma membrane | 0.58 | Glycosyl hydrolase 16 family |
| A0A1T5D928 | A0A1T5D928_9BACT | Glycosyl hydrolases family 16 | extracellular space | 0.96 | Glycosyl hydrolase 16 family |
| A0A1T5ER10 | A0A1T5ER10_9BACT | Pectinesterase (EC 3.1.1.11) | extracellular space | 0.96 |  |
| A0A1T5AKI5 | A0A1T5AKI5_9BACT | Glycosyl hydrolase family 115 | extracellular space | 0.97 | Glycosyl hydrolase 115 family |
| A0A1T5EVF8 | A0A1T5EVF8_9BACT | Glycosyl hydrolases family 28 | extracellular space | 0.96 | Glycosyl hydrolase 28 family |
| A0A1T5F929 | A0A1T5F929_9BACT | Glycosyl hydrolases family 43 | extracellular space | 0.91 | Glycosyl hydrolase 43 family |
| A0A1T5F9C9 | A0A1T5F9C9_9BACT | Glycosyl hydrolases family 43 | extracellular space | 0.84 | Glycosyl hydrolase 43 family |
| A0A1T5FMX4 | A0A1T5FMX4_9BACT | Beta-glucosidase | extracellular space | 0.8 | Glycosyl hydrolase 3 family |
| A0A1T5FN55 | A0A1T5FN55_9BACT | Beta-glucosidase | extracellular space | 0.91 | Glycosyl hydrolase 3 family |
| A0A1T5HSH3 | A0A1T5HSH3_9BACT | Alpha-N-arabinofuranosidase | extracellular space | 0.87 | Glycosyl hydrolase 51 family |
| A0A1T5HSZ7 | A0A1T5HSZ7_9BACT | Beta-glucosidase | cytoplasm | 0.7 |  |
| A0A1T5ERG3 | A0A1T5ERG3_9BACT | Beta-xylosidase | extracellular space | 0.96 | Glycosyl hydrolase 43 family |
| A0A1T5H7X4 | A0A1T5H7X4_9BACT | Beta-xylanase (EC 3.2.1.8) | extracellular space | 0.96 | Glycosyl hydrolase 10 (cellulase F) family |
| A0A1T5A2F6 | A0A1T5A2F6_9BACT | Glycosyl hydrolases family 16 | extracellular space | 0.96 | Glycosyl hydrolase 16 family |
| A0A1T5AAH0 | A0A1T5AAH0_9BACT | Beta-glucosidase | extracellular space | 0.91 | Glycosyl hydrolase 3 family |
| A0A1T5AIR1 | A0A1T5AIR1_9BACT | Beta-mannosidase | extracellular space | 0.93 | Glycosyl hydrolase 2 family |
| A0A1T5BDJ3 | A0A1T5BDJ3_9BACT | Por secretion system C-terminal sorting domain-containing protein | extracellular space | 0.94 | Glycosyl hydrolase 8 (cellulase D) family |
| A0A1T5BG53 | A0A1T5BG53_9BACT | Glycosidase | extracellular space | 0.92 | Glycosyl hydrolase 13 family |
| A0A1T5C858 | A0A1T5C858_9BACT | Glycosyl hydrolases family 2, TIM barrel domain | extracellular space | 0.79 | Glycosyl hydrolase 2 family |
| A0A1T5E1U6 | A0A1T5E1U6_9BACT | Cellulase (Glycosyl hydrolase family 5) | extracellular space | 0.89 | Glycosyl hydrolase 5 (cellulase A) family |
| A0A1T5F945 | A0A1T5F945_9BACT | Glycogen operon protein | cytoplasm | 0.7 | Glycosyl hydrolase 13 family |
| A0A1T5FVC9 | A0A1T5FVC9_9BACT | Alpha-amylase | cytoplasm | 0.7 | Glycosyl hydrolase 13 family |
| A0A1T5FTJ2 | A0A1T5FTJ2_9BACT | 1,4-alpha-glucan branching enzyme (EC 2.4.1.18) | cytoplasm | 0.7 | Glycosyl hydrolase 13 family, GlgB subfamily |
| A0A1T5H3K4 | A0A1T5H3K4_9BACT | Glycosyl hydrolases family 16 | cytoplasm | 0.7 | Glycosyl hydrolase 16 family |
| A0A1T5HTA4 | A0A1T5HTA4_9BACT | Mannan endo-1,4-beta-mannosidase (EC 3.2.1.78) | extracellular space | 0.97 | Glycosyl hydrolase 26 family |
| A0A1T5EWN6 | A0A1T5EWN6_9BACT | Mannan endo-1,4-beta-mannosidase | extracellular space | 0.95 | Glycosyl hydrolase 5 (cellulase A) family |
| A0A1T5AZ04 | A0A1T5AZ04_9BACT | Beta-glucosidase | cytoplasm | 0.7 | Glycosyl hydrolase 3 family |
| A0A1T5EX03 | A0A1T5EX03_9BACT | Por secretion system C-terminal sorting domain-containing protein | extracellular space | 0.97 | Glycosyl hydrolase 5 (cellulase A) family |
| A0A1T5F9G7 | A0A1T5F9G7_9BACT | Endoglucanase | cytoplasm | 0.7 | Glycosyl hydrolase 5 (cellulase A) family |
| A0A1T5F9N3 | A0A1T5F9N3_9BACT | Endoglucanase (EC 3.2.1.4) | extracellular space | 0.74 | Glycosyl hydrolase 9 (cellulase E) family |
| A0A1T5HLI1 | A0A1T5HLI1_9BACT | Cellulase (Glycosyl hydrolase family 5) | extracellular space | 0.86 | Glycosyl hydrolase 5 family |
| A0A1T5EQR5 | A0A1T5EQR5_9BACT | Glycosidase | extracellular space | 0.8 |  |
| A0A1T5BFZ4 | A0A1T5BFZ4_9BACT | Glycosyl hydrolases family 28 | plasma membrane | 0.72 | Glycosyl hydrolase 28 family |
| A0A1T5AAJ7 | A0A1T5AAJ7_9BACT | Acetyl esterase/lipase | extracellular space | 0.95 |  |
| A0A1T5ABF3 | A0A1T5ABF3_9BACT | Putative esterase | extracellular space | 0.96 |  |
| A0A1T5AJJ7 | A0A1T5AJJ7_9BACT | Acetyl esterase/lipase | cytoplasm | 0.7 |  |
| A0A1T5BSN2 | A0A1T5BSN2_9BACT | Acetyl esterase/lipase | extracellular space | 0.97 |  |
| A0A1T5D372 | A0A1T5D372_9BACT | Esterase/lipase | extracellular space | 0.8 |  |
| A0A1T5CCM6 | A0A1T5CCM6_9BACT | Glycosidase | cytoplasm | 0.7 |  |
| A0A1T5G9N6 | A0A1T5G9N6_9BACT | Acetyl esterase/lipase | cytoplasm | 0.7 |  |
| A0A1T5HSK0 | A0A1T5HSK0_9BACT | Peptidase family M23 | cytoplasm | 0.7 |  |
| A0A1T5CBB6 | A0A1T5CBB6_9BACT | Beta-glucosidase | plasma membrane | 0.87 | Glycosyl hydrolase 3 family |
| A0A1T5EQN5 | A0A1T5EQN5_9BACT | Polygalacturonase | extracellular space | 0.92 | Glycosyl hydrolase 28 family |
| A0A1T5ABU7 | A0A1T5ABU7_9BACT | Putative esterase | cytoplasm | 0.7 |  |
| A0A1T5HTR7 | A0A1T5HTR7_9BACT | Polysaccharide deacetylase | extracellular space | 0.93 |  |
| A0A1T5HSN3 | A0A1T5HSN3_9BACT | Endonuclease I | extracellular space | 0.77 |  |
| A0A1T5CB20 | A0A1T5CB20_9BACT | Pectate lyase | extracellular space | 0.96 | Polysaccharide lyase 1 family |
| A0A1T5DYD6 | A0A1T5DYD6_9BACT | Exo-beta-1,3-glucanase, GH17 family | extracellular space | 0.96 | Glycosyl hydrolase 17 family |
| A0A1T5HU42 | A0A1T5HU42_9BACT | Listeria/Bacterioides repeat-containing protein | extracellular space | 0.96 | Polysaccharide lyase 1 family |

**Table 4SM. Net charge of AsGH16 across pH range. Values were calculated using PDB2PQR v3.7.1: biomolecular structure conversion software (Dolinsky *et al.*, 2007; Olsson *et al.*, 2011; Jurrus *et al.*, 2018). Net charge is normalised to charge per 100 amino acids.**

| **pH** | **Net charge (unfolded)** | **Net charge (folded)** | **Net charge per 100 aa (unfolded)** | **Net charge per 100 aa (folded)** |
| --- | --- | --- | --- | --- |
| **0** | 57.99 | 57.90 | 6.65 | 6.64 |
| **1** | 57.87 | 57.10 | 6.64 | 6.55 |
| **2** | 56.75 | 52.51 | 6.51 | 6.02 |
| **3** | 47.00 | 36.22 | 5.39 | 4.15 |
| **4** | 2.85 | 1.99 | 0.33 | 0.23 |
| **5** | -52.03 | -39.74 | -5.97 | -4.56 |
| **6** | -71.75 | -63.98 | -8.23 | -7.34 |
| **7** | -80.77 | -74.69 | -9.26 | -8.57 |
| **8** | -84.51 | -80.98 | -9.69 | -9.29 |
| **9** | -88.88 | -85.94 | -10.19 | -9.86 |
| **10** | -108.18 | -95.72 | -12.41 | -10.98 |
| **11** | -132.74 | -112.64 | -15.22 | -12.92 |
| **12** | -145.12 | -131.30 | -16.64 | -15.06 |
| **13** | -160.95 | -149.59 | -18.46 | -17.15 |
| **14** | -167.10 | -161.69 | -19.16 | -18.54 |

**Table 5SM. Net charge of AsGH26 across pH range. Values were calculated using PDB2PQR v3.7.1: biomolecular structure conversion software (Dolinsky *et al.*, 2007; Olsson *et al.*, 2011; Jurrus *et al.*, 2018). Net charge is normalised to charge per 100 amino acids.**

| **pH** | **Net charge (unfolded)** | **Net charge (folded)** | **Net charge per 100 aa (unfolded)** | **Net charge per 100 aa (folded)** |
| --- | --- | --- | --- | --- |
| **0** | 44.99 | 44.52 | 12.39 | 12.26 |
| **1** | 44.94 | 42.69 | 12.38 | 11.76 |
| **2** | 44.4 | 38.08 | 12.23 | 10.49 |
| **3** | 39.75 | 27.99 | 10.95 | 7.71 |
| **4** | 19.35 | 11.4 | 5.33 | 3.14 |
| **5** | -5.7 | -6.03 | -1.57 | -1.66 |
| **6** | -16.06 | -15.99 | -4.42 | -4.40 |
| **7** | -23.89 | -22.54 | -6.58 | -6.21 |
| **8** | -27.38 | -25.97 | -7.54 | -7.15 |
| **9** | -30.23 | -28.02 | -8.33 | -7.72 |
| **10** | -40.08 | -31.6 | -11.04 | -8.71 |
| **11** | -53.96 | -39.82 | -14.87 | -10.97 |
| **12** | -61.77 | -49.62 | -17.02 | -13.67 |
| **13** | -71.62 | -59.84 | -19.73 | -16.48 |
| **14** | -75.44 | -67.76 | -20.78 | -18.67 |

**Table 6SM. Net charge of AsGH9 across pH range. Values were calculated using PDB2PQR v3.7.1: biomolecular structure conversion software (Dolinsky *et al.*, 2007; Olsson *et al.*, 2011; Jurrus *et al.*, 2018). Net charge is normalised to charge per 100 amino acids.**

| **pH** | **Net charge (unfolded)** | **Net charge (folded)** | **Net charge per 100 aa (unfolded)** | **Net charge per 100 aa (folded)** |
| --- | --- | --- | --- | --- |
| **0** | 51.99 | 51.94 | 9.38 | 9.38 |
| **1** | 51.93 | 51.42 | 9.37 | 9.28 |
| **2** | 51.31 | 48.19 | 9.26 | 8.70 |
| **3** | 45.98 | 38.46 | 8.30 | 6.94 |
| **4** | 22.57 | 19.39 | 4.07 | 3.50 |
| **5** | -5.56 | -2.26 | -1.00 | -0.41 |
| **6** | -15.98 | -14.85 | -2.88 | -2.68 |
| **7** | -21.87 | -21.92 | -3.95 | -3.96 |
| **8** | -24.81 | -26.85 | -4.48 | -4.85 |
| **9** | -29.74 | -31.95 | -5.37 | -5.77 |
| **10** | -49.87 | -41.32 | -9.00 | -7.46 |
| **11** | -77.4 | -55.34 | -13.97 | -9.99 |
| **12** | -88.98 | -69.93 | -16.06 | -12.62 |
| **13** | -98.8 | -84.77 | -17.83 | -15.30 |
| **14** | -102.47 | -94.72 | -18.50 | -17.10 |

**Figures**

**FIGURE 1SM.** **A.** Structure of glucuroxylan. Growth curves on glucuroxylan (**B**) and xylose (**C**), respectively. Negative control was performed with minimal media and carbon source alone, without *A. saponilacus*. **D.** PUL composition of PUL (predicted as xylan-active). **E.** RT-qPCR on SuS-C/SusD-like genes.

**FIGURE 2SM. A.** Activity of AsGH9 on potential substrates. **B.**  Activity of AsGH16 on potential substrates. **C.**  Activity of AsGH26 on potential substrates.

**FIGURE 3SM. A**. Structure of *As*GH16 with catalytic residues shown in zoom. **B.** Structure of *As*GH9 with catalytic residues shown in zoom. **C.** Structure of *As*GH26 with catalytic residues shown in zoom.

**FIGURE 4SM. A.** Structure of GH26 with surface charge. **B.** GH26 surface charge. **C.** Structure of GH9 with all CBMs. **D.** GH9 surface charge.
